# Single Shot Polarization Characterization of XUV FEL Pulses from Crossed Polarized Undulators

**DOI:** 10.1038/srep13531

**Published:** 2015-08-28

**Authors:** E. Ferrari, E. Allaria, J. Buck, G. De Ninno, B. Diviacco, D. Gauthier, L. Giannessi, L. Glaser, Z. Huang, M. Ilchen, G. Lambert, A. A. Lutman, B. Mahieu, G. Penco, C. Spezzani, J. Viefhaus

**Affiliations:** 1 Elettra-Sincrotrone Trieste, S.S. 14-km 163.5, 34149 Basovizza, Trieste, Italy; 2 Università degli Studi di Trieste, Dipartimento di Fisica, Piazzale Europa 1, 34127 Trieste, Italy; 3 European XFEL, 22761 Hamburg, Germany; 4 Laboratory of Quantum Optics, University of Nova Gorica, 5000 Nova Gorica, Slovenia; 5 Enea, via Enrico Fermi 45, 00044 Frascati, Roma, Italy; 6 DESY, 22607 Hamburg, Germany; 7 SLAC National Accelerator Laboratory, Menlo Park, California 94025, USA; 8 Stanford PULSE Institute, Menlo Park, CA, USA; 9 Laboratoire d’Optique Appliquée, ENSTA ParisTech-CNRS UMR 7639-Ecole polytechnique, Chemin de la Huniére, 91761 Palaiseau, France

## Abstract

Polarization control is a key feature of light generated by short-wavelength free-electron lasers. In this work, we report the first experimental characterization of the polarization properties of an extreme ultraviolet high gain free-electron laser operated with crossed polarized undulators. We investigate the average degree of polarization and the shot-to-shot stability and we analyze aspects such as existing possibilities for controlling and switching the polarization state of the emitted light. The results are in agreement with predictions based on Gaussian beams propagation.

The ability of modern high-gain free-electron lasers (FELs)^1^,^2^,^3^,^4^ to provide short and intense photon pulses in the extreme ultraviolet (XUV)—X-ray spectral range has opened new exciting opportunities in time-resolved and nonlinear experiments^5^,^6^,^7^. As with the case of synchrotron radiation sources and other XUV sources^8^,^9^,^10^ the possibility of controlling light polarization for FELs can be crucial for specific classes of experiment studying, e.g., the dynamics of magnetization, spectroscopy on chiral targets, or for polarization dependent spectroscopy. In synchrotrons, polarization control is achieved by using properly designed undulators that force the relativistic beam into a wiggling trajectory^11^,^12^,^13^,^14^.

While these solutions are nowadays widespread in modern synchrotron radiation facilities, they are not used in most of the operational and planned X-ray FELs. Among currently operational, FERMI is the only one equipped with variable polarization undulators^4^. LCLS is commissioning a variable polarization undulator to be used at the end of the main undulator line^15^. One reason favoring linearly polarized undulators over variable-polarization ones is their simplicity and lower cost. Furthermore, most variable polarization designs have difficulty in maintaining over lengths that easily exceed dozens of meters the high quality required at X-ray output wavelengths. The application of variable polarization undulators is therefore, for the moment, limited to FEL facilities based on shorter undulators. This is the case of seeded FELs, in which the amplification process is boosted by an external seeding source^16^. Another possibility, limited however to hard X-rays, is to use a properly designed phase retarder crystal to modify the polarization of the light^17^.

Alternative solutions for radiation polarization control in Self Amplified Spontaneous Emission (SASE) X-ray FELs have been proposed and studied in the last decades^18^,^19^,^20^,^21^. The most attractive possibility relies on the coherent superposition of two radiation pulses generated by orthogonally polarized undulators^19^. The crossed polarized undulator scheme has been demonstrated on a synchrotron source^22^ where polarized light has been successfully used for magnetic dichroism experiments^23^. More recently, the control of the polarization by means of a combination of undulators with different polarization states has also been reported in an FEL oscillator in the optical klystron configuration^24^. The effectiveness of the crossed polarized undulator in single pass FELs has been demonstrated only very recently with a dedicated experiment at the SINAP FEL test facility^25^. Such an experiment was carried out in the visible spectral range and in a low-gain FEL configuration.

We report here the first study of the crossed polarized undulators scheme on a high-gain seeded FEL operating in the XUV spectral range. Moreover, thanks to an experimental setup capable of measuring the degree of polarization on a singe shot basis, measurements of statistical fluctuations of the polarization properties have been done. This allowed us to characterize, also for the first time, the performance of the setup in terms of polarization reproducibility and stability, that is one of the main concerns when polarization is used for dedicated experiments^26^.

## Results

**Figure 1:**
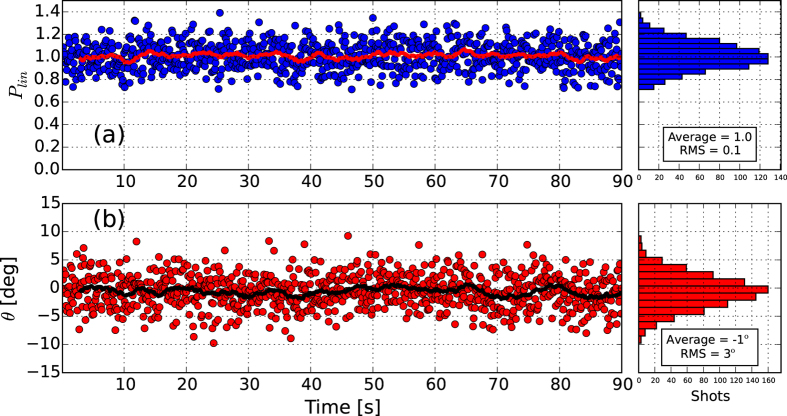
(**a**) Degree of linear polarization (*P*_*lin*_) and (**b**) direction of the polarization vector (*θ*) for the FEL produced when all the undulators are tuned to “pure” LH polarized light. The lines represent the moving average over 30 shots of the reported quantities. The histograms show the distribution of the data.

As a starting reference, we report in Fig. 1 a measurement done with all the six radiators tuned in the “pure” linear horizontal (LH) polarization mode. The degree of linearly polarized light (
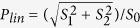
, *S* being the Stokes vector^27^) and the direction of the polarization vector (*θ* = 0.5*tan^−1^(*S*_2_/*S*_1_)) for each FEL shot are reported in Fig. 1(a,b) respectively, along with their histograms. The lines represent the moving average of *P*_*lin*_ and *θ* over 30 shots. The averages show that the light is characterized by a very high degree of horizontal polarization. The trend of the moving average shows the stability of the scheme. Single shot fluctuations both on the degree and the direction of polarization are dominated by the statistical measurement uncertainties that, as suggested by previous experiments^28^, greatly overcome the real fluctuations of the output light polarization. Reported fluctuations of 0.1 in the degree of polarization and 3° in the direction of polarization are determined by the accuracy of the measurement and not by real fluctuations of polarization properties. Accuracy is determined by fluctuations in single e-tof signals that have been operated in analogous current mode and may depend on the polarimeter settings. Data with a degree of linear polarization that exceeds one is physically not possible but algebraically correct for the used model as imprinted in the statistical fluctuations on the e-tof signal (see Fig. 1).

**Figure 2:**
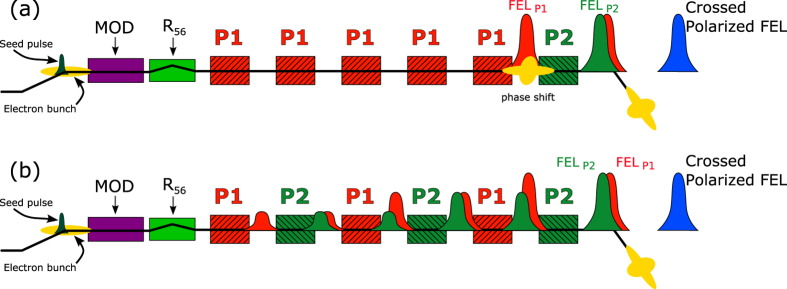
Schematic representation of the FEL-1 setup. The crossed polarized undulator scheme can be implemented as a superposition of two consecutives sources (**a**) or in a distributed scheme (**b**). Red and green represent undulators with two orthogonal polarizations (P1 and P2) that can be either CR - CL, or LH - LV that produce FEL pulses with corresponding polarization properties (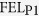 and 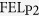). MOD is the modulator undulator, R56 is the dispersive section (see Methods).

When the undulator is configured in crossed polarized undulator mode (see Methods) as in Fig. 2(a,b), the setting of the phase shifters is extremely critical. By controlling the light-electron phase in different undulators, one can indeed tune the polarization state of the final FEL pulse. In order to obtain a pure polarization state the two superimposing fields must have the same intensity and constant phase relation between each other.

**Figure 3:**
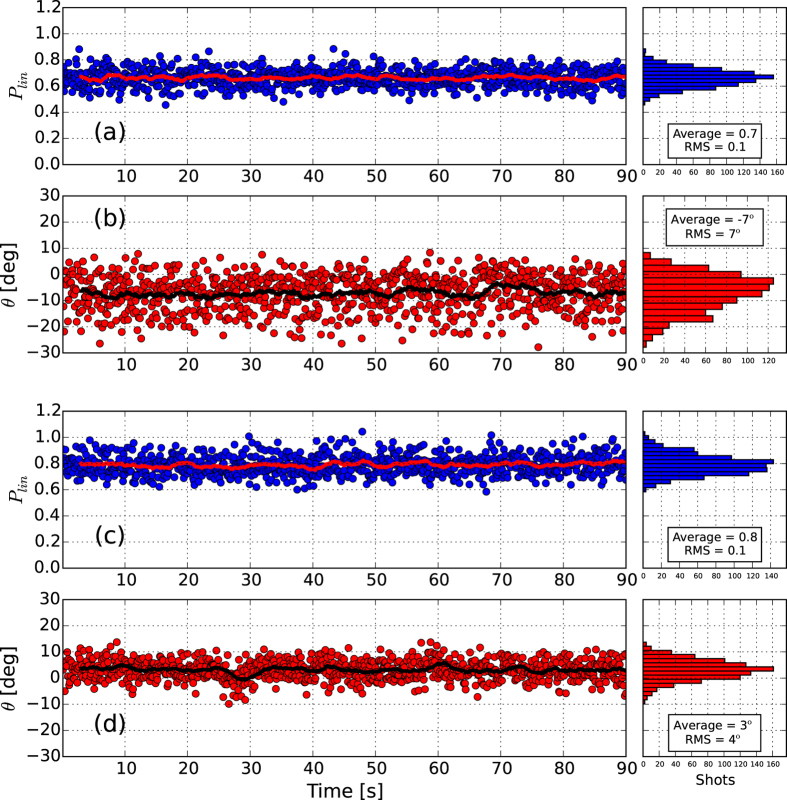
Degree and direction of the linear polarization component for the crossed circularly polarized undulators. (**a**) and (**b**) are relative to the consecutive scheme (Fig. 2(a)), while (**c**) and (**d**) refer to the distributed scheme (Fig. 2(b)). The lines represent the moving average over 30 shots of the reported quantities.

In Fig. 3, results for the crossed polarized undulator setup are shown for the case that uses circular left (CL) and circular right (CR) polarized radiation superimposed to generate linear horizontal (LH) light. The degree of polarization is reported both for the consecutive and the distributed crossed polarized undulator setups (respectively Fig. 3(a,c), see Methods). The average degree of linear polarization is 70% in the first case, and 80% in the latter. Figure 3 also reports the direction of the polarization vector as a function of time for the two studied undulator configurations (Fig. 3(b,d)). In both cases the direction is compatible with the expected LH direction and the small tilt with respect to a perfect horizontal polarization (*θ* = 0) is associated to the setting of the phase shifter.

It is worth noting that the fluctuations of the polarization direction are different for the consecutive and distributed scheme. While in the case of the alternate undulator scheme the measured fluctuations are within the accuracy of the instrument as for the “pure” polarization of Fig. 1, in the case of consecutive crossed undulators fluctuations on the direction of polarization *θ* are significantly larger than the instrumental accuracy. This indicates that direction of polarization is changing shot to shot as a consequence of the fluctuation on e-beam and FEL parameters. Such a results is originated by the different sensitivity to FEL power in the two orthogonal undulator groups using the consecutive and alternate undulator schemes. Effects on the degree of polarization *P*_*lin*_ can not be measured, hence indicating, to our understanding, that they are smaller than the instrumental accuracy. In terms of average polarization parameters, both configurations are able to produce a high degree of horizontally polarized light, although lower than the one obtained in pure linear mode. A clear benefit in using the distributed scheme is the higher stability of the polarization parameters of the produced radiation.

**Figure 4:**
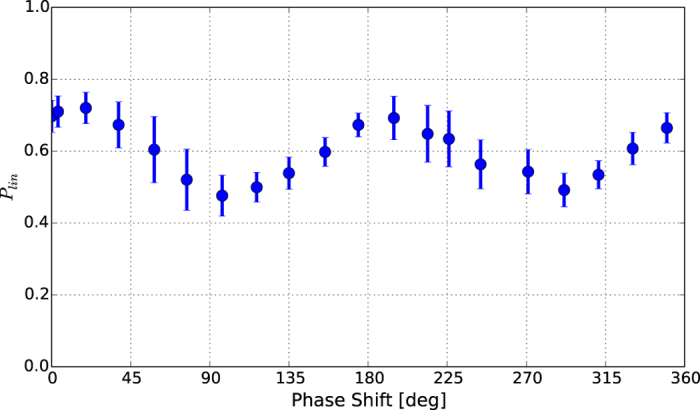
Degree of linear polarization *P*_*lin*_ as a function of the phase shift between the two undulator groups, when LH and LV polarization are composed in the crossed polarized undulator scheme. The dots display the individual shot-to-shot measurements for each given phase shift with the measured errorbar computed respectively as the average and RMS of the acquired data for each value of the phase shift.

One of the driving motivations for studying the crossed polarized undulator scheme is to demonstrate the capability of high-gain FEL’s to change the state of the output polarization of the light by simply tuning the phase shifters installed between the undulators. Such a capability would be of great interest for a number of applications relying on dichroic effects. In the following, we focus on the consecutive undulator arrangement (Fig. 2(a)), but similar results can be obtained with the distributed scheme (Fig. 2(b)) if proper phase shifter tuning is applied^29^. The degree of linear polarization measured for the LH and LV crossed polarized undulator is reported in Fig. 4 as a function of the phase shift introduced between the two consecutive groups. This set of measurements has been obtained with slightly different polarimeter settings that, by increasing the e-tof signal, improve the accuracy for the degree of polarization to 0.05 with respect to 0.1 of the settings used in the measurements reported in Figs 1 and 3. By changing the phase shifter, one can, in principle, change the output radiation from linear (*P*_*lin*_ = 1), at phase shifts of *π*, to circular (*P*_*lin*_ = 0), if a 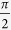 phase shift is introduced. The maximum and minimum measured degree of linear polarization are respectively 70% and 45%.

**Figure 5:**
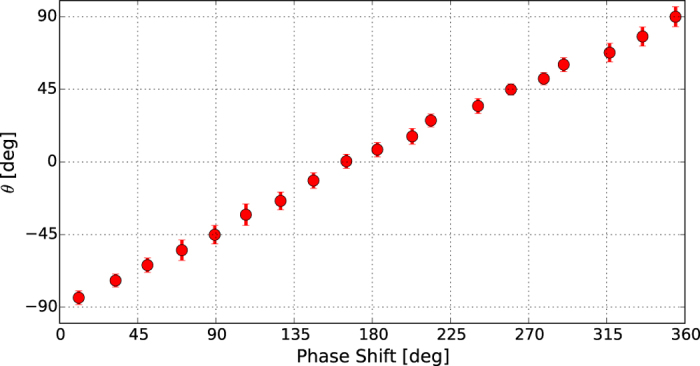
Direction of linear polarization *θ* as a function of the phase shift between two undulator groups, when CL and CR radiations are composed to realize the crossed polarized undulator scheme.

Finally, in Fig. 5 we report the results of an experiment confirming the possibility of controlling the direction of the linear polarization by varying the phase between the two consecutive sets of undulators tuned to emit CR and CL polarized light. The direction of the linear polarization component has a linear dependence with respect to the phase shift in between the two sets of undulators and continuously varies between −90 and +90 degrees, corresponding to a full rotation of the polarization vector direction.

## Discussion

The results presented above demonstrate that the crossed polarized undulator scheme has been successfully implemented at FERMI in the XUV wavelength range, in both a consecutive and a distributed undulator scheme. Better performance, with a maximum degree of linear polarization of 80% has been measured with the distributed scheme. The stability of both schemes has been investigated, both in terms of direction of the polarization vector and degree of linear polarization.

Because the two orthogonal fields have different source points, only the on-axis phase can be adjusted to the desired value to define the final polarization: the relative phase and intensity of off-axis fields change with the distance from the axis. As a result, different polarization states will be produced off-axis. This leads to a reduced degree of polarization. This effect can be estimated by using simple formulas for Gaussian beams^30^ where it can be shown that the key parameter is the ratio of the Rayleigh length of the emitted radiation over the distance between the two sources’ positions, the latter being identified with the centers of the two orthogonal undulator sets^31^. If this ratio is much larger than one the light emitted from one undulator will not diverge significantly when superimposed to the second source point, so the total output radiation could in principle be nearly 100% polarized. If instead the ratio is smaller than one, the off-axis radiation emitted from the first source will interfere at different phases with the orthogonal light emitted by the second source, leading to a reduced degree of polarization as described above.

In the case of the reported experiment with the consecutive scheme (Fig. 2(a)), the distance between the source points of the two fields correspond to the distance between the centers of the last two undulators (3.7 m) that is significantly larger than the Rayleigh range (2.2 m). This gives a total degree of linear polarization of ∼70% when summing two Gaussians beam. This result is in quite good agreement with data reported in Fig. 3(a). As already proposed elsewhere^29^,^31^,^32^, by adopting the distributed scheme (Fig. 2(b)) the divergence effects can be mitigated. The same model predicts a maximum degree of polarization of 80% in the case of the parameters reported in Table 1. The good agreement with theoretical predictions of this simple model in terms of degree of polarization with crossed polarized undulators is an indication of the high degree of coherence of the two FEL pulses that are interfering to generate the polarized light. The model is also capable of describing the limit in the generation of circular polarization in the phase shifter scan (Fig. 4) via the wavefront curvature together to a slight unbalance in the intensity of the fields emitted by the two undulator groups.

The fact that the degree of linear polarization does not reach 100% suggests that a non negligible amount of unpolarized light is produced. The Stokes parameters predicted by the model have a radial dependency, i.e. different parts of the wavefront have different polarization properties, leading to an overall loss of polarization. Nevertheless the measured levels of polarization would be sufficient for a wide range of experiments requiring polarization control. This also suggests that, in case a larger degree of polarization is required, limiting the collection aperture of the radiation could further increase the output degree of polarization of the light produced via the crossed undulator scheme.

Shot-to-shot fluctuations in the degree of polarization obtained with crossed polarized undulators have been measured to be comparable to what obtained with the purely polarized undulator and being lower than the instrumental noise. An increase of a factor ∼2 in the fluctuations of the direction of polarization has been observed for the consecutive crossed polarized undulator scheme. Such a result is related to the larger fluctuations for the relative power of the two orthogonal fields in this undulator configuration where the FEL gain is occurring mainly in one of the two undulator subsets.

The good agreement between the measured degrees of linear polarization and theoretical predictions indicates a high degree of coherence of the pulses produced by the two undulator groups. The achieved performance in terms of polarization control at FERMI can be used to extend the present capability of the two FERMI FELs to generate linearly polarized radiation with arbitrary direction, as indicated in Fig. 5. The use of crossed polarized undulators has the interesting feature of allowing rapid switching between two polarization states by using a fast phase shifter placed between the two orthogonally polarized undulators. For upcoming X-FELs, the use of an electromagnetic phase shifter could allow in the future such switching on kHz or faster repetition rates. This capability might be useful to improve experimental signal to noise ratios by strongly reducing sensitivity to slow timescale (e.g. ≥1 sec.) drifts in the FEL output properties.

## Methods

### Machine setup

FERMI is a seeded FEL based on the HGHG scheme^16^ single stage for FEL-1^33^ and double-stage for FEL-2^34^. The reported experiment has been performed on FEL-1 operated at 32 nm, corresponding to the 8^*th*^ harmonic of the 260 nm seed laser. Through the interaction with the seed laser in the modulator (MOD) a bunching at the desired wavelength is produced into the electron beam that generates the coherent emission in the forthcoming radiator. The bunching amplitude can be controlled via a dispersive section magnet (*R*_5,6_). The aforementioned beam is produced in a photo-cathode gun^35^ and accelerated and manipulated in a normal conducting RF linac^36^ up to an energy of 1.22 GeV and a current of 600 A over ∼1 ps in the case of the described experiment. Additional details including the electron beam and seed laser parameters used in this experiment can be found in Ref. 28.

### Undulator schemes

Each of the six undulators defining the FEL-1 radiator, can be independently tuned in wavelength and polarization allowing various schemes for crossed polarized undulators. One of the key design features of FERMI is the choice of variable polarization, APPLE-II type undulators^14^, which can be tuned to emit FEL light with linear horizontal (LH), linear vertical (LV) and circular (with left and right chirality, CL and CR respectively) polarization^28^. In each undulator break phase shifters are installed. These are small permanent magnet chicanes that are used to keep electrons in phase with the light pulse emitted by the different undulator sections.

Two approaches are possible at FERMI to implement the crossed polarized undulator scheme; the first relies on the superposition of two orthogonal linearly polarized undulators to produce circular polarization. Alternatively, two orthogonal circularly polarized undulators (i.e., counter-rotating) can be used to generate linear polarization. Moreover, the flexibility of the FERMI undulator setup allows one to use two undulator configurations as illustrated in Fig. 2(a,b). Similarly to the original proposal^19^, one can implement the crossed polarized undulator scheme by setting a first group of undulators to a given polarization state (CR or LH), and a second set of undulators to the orthogonal polarization (CL or LV). In the case of an FEL characterized by an exponential growth of the power along the radiator, one requires an unbalanced number of undulators for the two sets. For FERMI the required conditions can be obtained with 5 undulators for the first group and one for the second as indicated in Fig. 2(a).

Another possibility, see Fig. 2(b), is to implement the distributed crossed polarized undulator scheme already discussed in^29^,^32^, in which the undulators are tuned with alternating polarization. In this case both orthogonal fields will exponentially grow on the two undulator groups that can, therefore, have the same number of undulators. At FERMI, emission power from the two orthogonal undulators can be independently adjusted by setting seeding parameters^31^.

A schematic representation of the undulator system and of the seeding scheme is shown in Fig. 2; the main parameters are reported in Table 1.

### e-TOF spectrometer

In order to characterize the polarization state of the emitted radiation, a 16 channel time-of-flight electron spectrometer (e-tof)^37^ was installed at the back of the FERMI DiProi beamline^38^. Here measurements of the angular distribution of photoelectrons produced by the FEL pulse passing through an atomic gas target are used for reconstructing, on a shot-to-shot basis, the polarization state of the FEL XUV radiation. The instrument is capable of fully characterizing the degree and the direction of the linear component of the polarization. The circular polarization component is instead characterized by an angular distribution of the photoelectrons that is indistinguishable from the unpolarized componet. If one assume that the unpolarized component is negligible^28^ (*S*_0_ = 1), the circular polarization component (*S*_3_) can be inferred from the linear polarization degree (*P*_*lin*_) via a quadrature difference as 
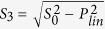
.

## Additional Information

**How to cite this article**: Ferrari, E. *et al*. Single Shot Polarization Characterization of XUV FEL Pulses from Crossed Polarized Undulators. *Sci. Rep*. **5**, 13531; doi: 10.1038/srep13531 (2015).

## Figures and Tables

**Table 1:** Relevant parameters for the FEL configuration used in the experiment.

Undulator length	6 × 2.4 m
Break length	1.3 m
FEL wavelength	32 nm
FEL source size (rms)	150 *μ*m
FEL Rayleigh range	2.2 m
FEL pulse energy	50 *μ*J
